# Corrigendum to “Myricetin Possesses Potential Protective Effects on Diabetic Cardiomyopathy through Inhibiting I*κ*B*α*/NF*κ*B and Enhancing Nrf2/HO-1”

**DOI:** 10.1155/2021/9812928

**Published:** 2021-05-04

**Authors:** Hai-han Liao, Jin-xiu Zhu, Hong Feng, Jian Ni, Nan Zhang, Si Chen, Huang-jun Liu, Zheng Yang, Wei Deng, Qi-Zhu Tang

**Affiliations:** ^1^Department of Cardiology, Renmin Hospital of Wuhan University, Wuhan, China; ^2^Cardiovascular Research Institute of Wuhan University, Wuhan, China; ^3^Hubei Key Laboratory of Cardiology, Wuhan, China; ^4^Department of Gerontology, Renmin Hospital of Wuhan University, Wuhan 430060, China

In the article titled “Myricetin possesses potential protective effects on diabetic cardiomyopathy through inhibiting I*κ*B*α*/NF*κ*B and enhancing Nrf2/HO-1” [1], an error was identified in Figure 8(b) where the same original blots were mistakenly used for T-Nrf2 and HO-1 when preparing the image. The authors have provided the correct images for T-Nrf2 and HO-1 and the revised Figure 8 is as below:

## Figures and Tables

**Figure 1:**
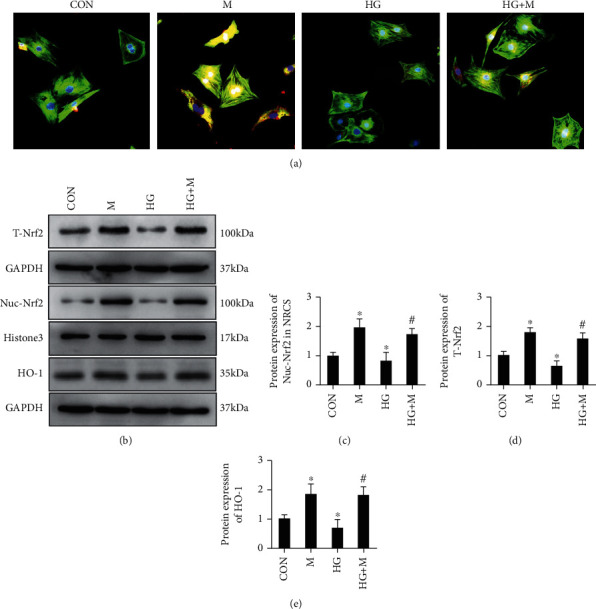
Myricetin regulated the expression of Nrf2 and HO-1 in neonatal rat cardiomyocytes (NRCM). (a) Myricetin enhanced the expression and nuclei translocation of Nrf2 after 36 h treatment with or without high glucose. (b) Representative blots showed the expression of Nrf2 and HO-1. (c–e) Histogram showed the fold change of T-Nrf2, Nuc-Nrf2, and HO-1; all of the proteins were normalized to GAPDH or histone 3 before relative quantitative analysis, and all experiments were repeated 3 times independently. Data were presented as means ± SD. ^∗^*P* < 0.05 as compared with CON, and #*P* < 0.05 as compared with HG group.
